# Xanthate Based Radical Cascade Toward Multicomponent Formation of Pyrrolopyrimidines

**DOI:** 10.3390/molecules16119261

**Published:** 2011-11-04

**Authors:** Laurent El Kaïm, Laurence Grimaud, Patil Pravin

**Affiliations:** Laboratoire DCSO ENSTA-Polytechnique-CNRS, UMR 7652, Ecole Nationale Supérieure de Techniques Avancées, 32 Bd Victor, 75739 Paris Cedex 15, France

**Keywords:** Ugi-Smiles coupling, xanthates, multicomponent reactions, radical processes, isocyanides

## Abstract

A short sequential synthesis of pyrrolidino- pyridines and pyrimidines illustrates the potential of combining Ugi-Smiles couplings with radical tin-free processes.

## 1. Introduction

**Scheme 1:**
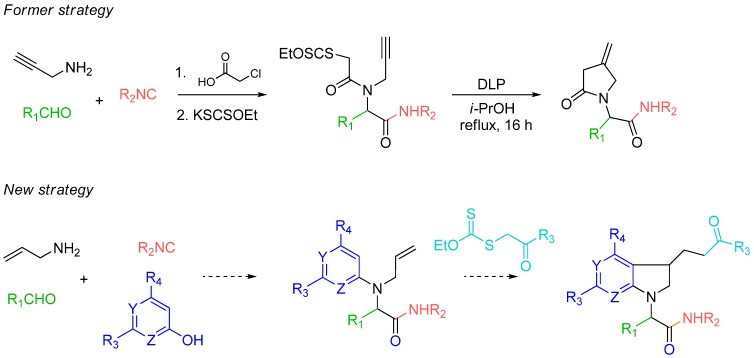
Synthetic strategy towards pyrrolidino fused systems.

Among multi-component reactions (MCRs), the Ugi reaction has a privileged position due to its broad synthetic significance [1,2]. Its four points of diversity combined with possible further transformations of the resulting adducts have became very popular in heterocyclic synthesis. Indeed, the Ugi reaction has been associated with a wide range of post-condensation reactions in order to form even more complex scaffolds, including cycloadditions [3,4,5,6,7,8,9], metal-catalyzed processes [10,11,12,13,14,15] and cyclocondensations [16,17,18,19,20,21]. Among this very impressive bibliography, the combination of Ugi or Passerini reactions with radical chemistry has been scarcely documented [22,23,24,25,26,27]. Among the various radical systems explored in our group, we have mostly studied the potential of xanthate radical transfer resulting in various cyclizations of Ugi xanthate adducts bearing suitable alkene [22], alkyne [25] or aryl [26] moieties. In all these examples, α-chloroacetic acid was used as the acidic partner to introduce the xanthate moiety via a nucleophilic substitution. However, these intramolecular post-condensations did not exploit the full potential of the xanthate radical transfer. Indeed, compared to tin hydride chemistry, the reversible nature of the addition of radicals onto the thiocarbonyl group is associated with high yielding intermolecular couplings between xanthates and alkenes [28,29]. Herein, we propose to explore this feature and to further increase the molecular diversity of the Ugi step through a xanthate triggered addition/cyclization cascade to form new heterocyclic systems (Scheme 1).

## 2. Results and Discussion

**Scheme 2:**
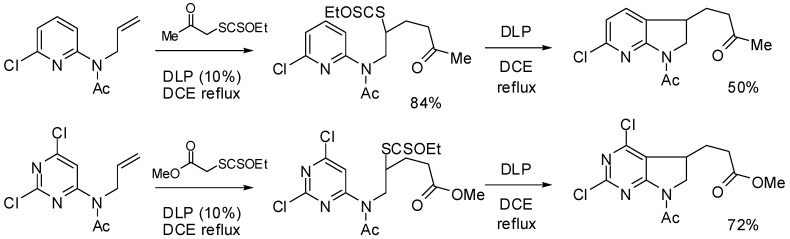
Selected examples from Zard’s group [31,33].

Developing the radical chemistry of xanthates, the Zard group has reported various synthetic approaches towards fused cyclic systems such as indanes, indolines or azaindolines [30,31,32,33,34,35]. These syntheses feature an intermolecular addition of a xanthate on an alkene followed by an intramolecular trapping with a suitably positioned aryl group. As part of our ongoing interest in Ugi-type reactions, we naturally imagined to synthesize the radical acceptor through a Ugi-Smiles coupling. Indeed, we recently developed the use of electron-deficient phenols as acid surrogates in Ugi reactions to form *N*-arylamino carboxamides [36,37]. The use of a partner substituted with an alkene, such as allylamine, in this reaction would afford in one step an interesting substrate to test the tandem radical process (Scheme 1). Concerning the phenol, most reported Ugi-Smiles couplings involve a nitro group to perform the Smiles rearrangement. As nitroaryl derivatives are often associated with inhibition of radical chain processes, we prefer the use of hydroxy heterocycles (pyridines, pyrimidines) which also led to efficient multicomponent couplings [38]. *N*-allylaminopyridines and *N*-allylaminopyrimidines are known to react with xanthate under radical conditions, however the scope of these reactions seems to be limited by the nucleophilic behavior of the cyclic nitrogen atom. Indeed all reported radical tandem were performed on 2- or 5-halosubstituted heterocycles (chloro and fluoro) in order to lower the reactivity at the nitrogen center (Scheme 2) [31,32,33,34,35].

### 2.1. Four-Component Coupling Towards Radical Substrates

Various Ugi-Smiles adducts were thus prepared starting with allylamine as the amine partner. The latter was coupled with different hydroxy- pyridines and pyrimidines. The MCR coupling was performed under classical conditions using methanol as solvent. The starting radical substrates were obtained in modest to good yields.

**Table 1:** Preparation of the radical substrates. 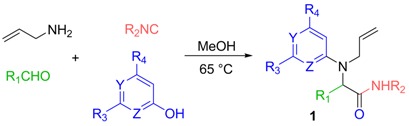

Entry	R_1_	R_2_	R_3_	R_4_	Y	Z	Product (%)
1	Et	Cy	Me	Me	N	N	**1a** (38%)
2	*i*-Bu	Cy	H	H	N	CH	**1b** (69%)
3	*i*-Bu	*t-*Bu	H	H	N	CH	**1c** (43%)
4	Et	Cy	Ph	Me	N	N	**1d** (61%)
5	H	*p*-MeBn	Ph	Me	N	N	**1e** (60%)
6	Et	Cy	H	H	CNO_2_	N	**1f** (41%)
7	*i*-pr	*p*-ClBn	Me	Me	N	N	**1g** (68%)

### 2.2. Radical Cascade

**Scheme 3:**
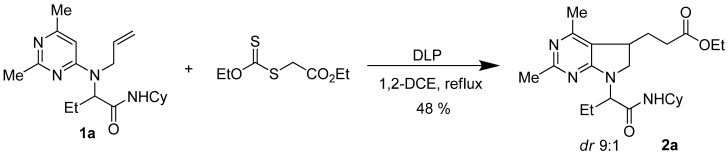
Radical reaction conditions.

In previous studies on xanthate additions on *N*-allylamino- pyridines and pyrimidines, a first 1,2-addition product on the alkene could be obtained and isolated under heating with substoichiometric amounts of a radical initiator such as dilauroyl peroxide (DLP) in refluxing 1,2-dichloroethane (DCE) or ethyl acetate. Then the intermediate adducts could cyclize under treatment with further DLP or di-*tert-*butyl peroxide (more than 1 equivalent) at higher temperature allowing cleaner reactions (Scheme 3). Even if the yields are expected to be lower, we preferred to perform the cascade under a single set of experimental conditions in order to keep the multicomponent sequence as short as possible. When adduct **1a** was refluxed in DCE (0.2 M) with DLP added in 15 mol% portions every twenty minutes, we could isolate the desired cyclized pyrrolidine **2a** as a 9:1 mixture of diastereomers [39] in 48% isolated yield, after overall addition of 1.5 equivalent of DLP (Scheme 3).

**Scheme 4:**
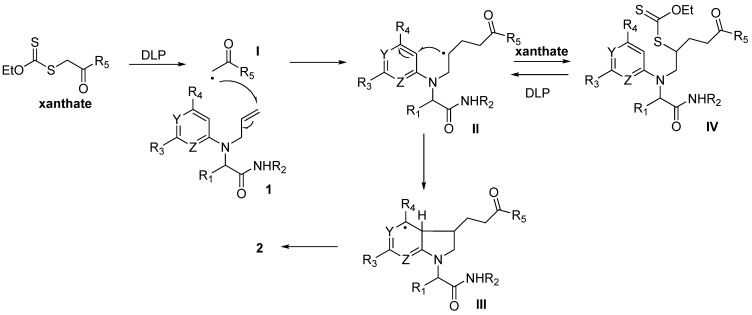
Plausible mechanism.

This sequence is initiated by decomposition of the xanthate into the electrophilic radical **I**, which is then trapped by the alkene moiety to give intermediate **II**. The latter is an electron-rich radical that can react with the starting xanthate to form intermediate **IV**. The latter being slightly less reactive than the starting xanthate towards radicals issued from DLP, accumulates in the medium and reacts when further amount of DLP is added without much **1** left to enter in the chain process. After addition of the radical onto the aryl ring, the intermediate species **III** further aromatize through interaction with the DLP (Scheme 4).

To evaluate the scope of this cyclization, the reaction was performed with the Ugi-Smiles adducts formerly prepared and the results are displayed in Table 2.

**Table 2:** Radical additions-Cyclizations. 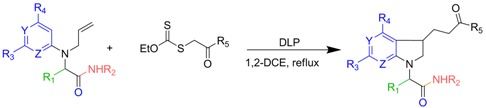

Entry	Ugi-Smiles adduct	Xanthate	Cyclized product	Yield (%)
1	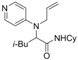 **1b**	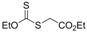	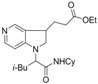 **2b** (*dr* 4:1)	24
2	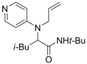 **1c**	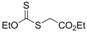	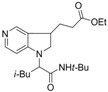 **2c** (*dr* 2:1)	27
3	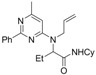 **1d**	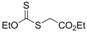	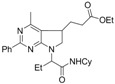 **2d** (*dr* 2.3:1)	55
4	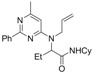 **1d**	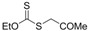	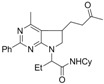 **2e** (*dr* 1.5:1)	44
5	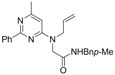 **1e**	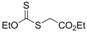	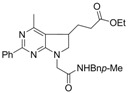 **2f**	27
6	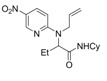 **1f**	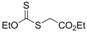	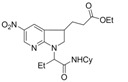 **2g** (*dr* 3:1)	48
7	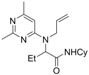 **1a**	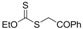	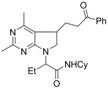 **2h** (*dr* 4:1)	34
8	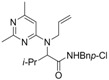 **1g**	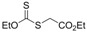	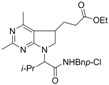 **2i** (*dr* 2.3:1)	43

Pyridines and pyrimidines without any halogen substitutent at the 2 or 5 position can act as radical trapping agents in this sequence. In the study disclosed by Zard, initial attempts with 2-aminopyridine or 2-amino-5-methylpyridine as starting materials led to successful 1,2-addition product with the xanthate, but no indolizine could be formed in the following cyclization step [31]. Chloro or fluoro substituents are expected to lower the electron density in the aromatic ring leading to easier addition of the nucleophilic intermediate radical species, and this may explain the moderate yields observed. In our case, the reaction turns out to be poorly efficient with 4-aminopyridines, with yields not exceeding 30% (Table 2, Entries 1–2), a nitro group at the 5-position of the 2-aminopyridine ring activates the cyclization as a higher 48% isolated yield could be obtained (Table 2, Entry 6). Pyrimidines, in agreement with their higher electrophilicity, are better partners, giving yields over 50% with simple alkyl or aryl susbtituents on the heterocyclic core (Table 2, Entry 3).

## 3. Experimental

### 3.1. General

^1^H-NMR spectra were recorded on a 400 MHz spectrometer (Bruker Avance 400), using CDCl_3 _solvent as reference and/or internal deuterium lock. ^13^C-NMR spectra were recorded on a 100.6 MHz spectrometer (Bruker Avance 400). Two-dimensional NMR spectroscopy [^1^H -^1^H COSY spectra, ^1^H - ^13^C COSY spectra (HSQC) and long-range ^1^H -^13^C COSY spectra (HMBC)], were carried out to determine the correlation between ^1^H and ^13^C. The chemical shifts for all NMR spectra are expressed in parts per million to high frequency of TMS reference. Coupling constants (*J*) are quoted in Hz and are recorded to the nearest 0.1 Hz. The IR spectra were obtained using ATR accessories on a Bruker Tensor 27 instrument. High-resolution (HR) mass spectra were recorded on a *Hewlett-Packard HP 5989B *spectrometer. TLC was carried out using precoated plates of silica gel 60F254 (SDS-Carlo-Erba).

### 3.2. Ugi-Smiles Coupling Procedure

To a 1 M solution of hydroxyheteroaromatic compound in methanol were added successively 1.0 equivalent of amine, 1.0 equivalent of aldehyde and 1.0 equivalent of isocyanide. The resulting mixture was stirred at 65 °C for 18–36 h. The solvent was removed afterwards under reduced pressure to afford the Ugi-Smiles product after purification by flash chromatography on silica gel.

### 3.3. DLP-Cyclisation Reaction Procedure

A solution of Ugi-Smiles adduct (1.0 mmol) and xanthate (1.2 mmol) in 1,2-dichloroethane (1.0 M) was reﬂuxed for 5 min under argon before DLP was added (60 g, 0.15 mmol) from the top of the condenser. Portions of DLP (60 mg, 0.15 mmol) were added every 20 min until complete disappearance of Ugi-Smile adduct and the starting xanthate, then the remaining DLP was added every 20 min until complete disappearance of the intermediate (TLC monitoring). Concentration under reduced pressure aﬀorded an oily pale brown residue, which was purified by ﬂash chromatography eluting with petroleum ether/diethyl ether to give the pure product as a mixture of two diastereomers. *In all the cases, the spectral data are given for the major diastereomer.*

*2-(N-Allyl-N-(2,6-dimethylpyrimidin-4-yl)amino)-N-cyclohexylbutanamide *(**1a**). Yellow solid, (1.0 gm, 38%), mp 124–125 °C; ^1^H-NMR: δ (ppm) 6.69 (br s, 1H), 6.11 (s, 1H), 5.86–5.69 (m, 1H), 5.27–5.01 (m, 3H), 4.08 (dd, 1H, *J* = 4.8, 17.0 Hz), 3.84 (d, 1H, *J* = 17.0 Hz), 3.75–3.63 (m, 1H), 2.51 (s, 3H), 3.32 (s, 3H), 2.13–2.00 (m, 1H), 1.92–1.81 (m, 1H), 1.78–1.59 (m, 3H), 1.58–1.46 (m, 2H), 1.40–1.22 (m, 2H), 1.20–1.06 (m, 2H), 1.02–0.93 (m, 1H), 0.90 (t, 3H, *J* = 7.3 Hz); ^13^C-NMR: δ (ppm) 170.1, 166.1, 165.3, 162.6, 133.5, 116.9, 100.3, 58.8, 47.6, 47.3, 32.9, 32.7, 26.1, 25.5, 24.5, 24.4, 24.3, 21.9, 10.9; IR (thin film): 2929, 2855, 2356, 1657, 1583, 1539, 1479, 1448, 1403, 1343, 1273, 1177, 1089 cm^−1^; HRMS: Calcd. for C_19_H_30_N_4_O: 330.2420, Found: 330.2425.

*2-(N-Allyl-N-(pyridin-4-yl)amino)-N-cyclohexyl-4-methylpentanamide *(**1b**). Pale yellow solid, (475 mg, 69%), mp 110–111 °C, ^1^H-NMR: δ (ppm) 8.23 (dd, 2H, *J* = 1.6, 5.2 Hz), 6.59 (dd, 2H, *J* = 1.6, 5.2 Hz), 5.95–5.80 (m, 2H), 5.29–5.21 (m, 2H), 4.25 (t, 1H, *J* = 6.9 Hz), 4.00 (dd, 1H, *J* = 5.2, 17.2 Hz), 3.90 (dd, 1H, *J* = 5.2, 17.2 Hz), 3.80–3.69 (m, 1H), 2.08–2.00 (m, 1H), 1.86–1.76 (m, 2H), 1.70–1.53 (m, 5H), 1.39–1.25 (m, 2H), 1.15–1.00 (m, 3H), 0.92 (d, 3H, *J* = 6.7, Hz), 0.90 (d, 3H, *J* = 6.7 Hz); ^13^C-NMR: δ (ppm) 169.9, 153.0, 150.2, 133.2, 118.1, 108.3, 61.0, 49.7 , 48.4, 37.7, 32.9, 25.4, 25.2, 24.7, 22.9, 22.1; IR (thin film): 2930, 2853, 1652, 1596, 1544, 1513, 1450, 1235, 1169 cm^−1^; HRMS: Calcd. for C_20_H_31_N_3_O: 329.2467, Found: 329.2467.

*2-(N-Allyl-N-(pyridin-4-yl)amino)-N-tert-butyl-4-methylpentanamide* (**1c**). Pale yellow solid, (360 mg, 43%), mp 111–112 °C, ^1^H-NMR: δ (ppm) 8.25 (dd, 2H, *J* = 1.6, 5.0 Hz), 6.59 (dd, 2H, *J* = 1.6, 5.0 Hz), 5.90–5.80 (m, 2H), 5.28–5.19 (m, 2H), 4.19 (dd, 1H, *J* = 5.9, 8.0 Hz), 4.00 (ddt, 1H, *J* = 1.5, 5.0, 17.2 Hz), 3.89 (ddt, 1H, *J* = 1.5, 5.0, 17.2 Hz), 2.04–1.95 (m, 1H), 1.65–1.53 (m, 2H), 1.27 (s, 9H), 0.91 (d, 3H, *J* = 6.6 Hz), 0.89 (d, 3H, *J* = 6.6 Hz); ^13^C-NMR: δ (ppm) 170.0, 153.0, 149.8, 133.1, 117.9, 108.3, 61.4, 51.4, 49.6, 37.5, 28.5, 25.0, 22.8, 22.2; IR (thin film): 2964, 2930, 2871, 1676, 1596, 1544, 1513, 1454, 1367, 1266, 1231, 1172 cm^−1^; HRMS: Calcd. for C_18_H_29_N_3_O: 303.2311, Found: 303.2309.

*2-(N-Allyl-N-(6-methyl-2-phenylpyrimidin-4-yl)amino)-N-cyclohexylbutanamide* (**1d**). white solid, (600 mg, 61%), mp 114–115 °C, ^1^H-NMR: δ (ppm) 8.50–8.27 (m, 2H), 7.60–7.38 (m, 3H), 6.60 (br s, 1H), 6.24 (s, 1H), 5.94–5.76 (m, 1H), 5.42–5.03 (m, 3H), 4.11 (d, 1H, *J* = 17.0 Hz), 3.96 (d, 1H, *J* = 17.0 Hz), 3.77–3.61 (m, 1H), 2.45 (s, 3H), 2.24–2.08 (m, 1H), 1.89–1.74 (m, 2H), 1.68–1.36 (m, 4H), 1.33–1.13 (m, 2H), 1.04–0.80 (m, 6H); ^13^C-NMR: δ (ppm) 170.2, 166.0, 162.8, 162.6, 138.2, 133.5, 130.2, 128.4, 127.9, 117.1, 101.3, 59.7, 47.7, 32.8, 32.6, 25.3, 24.6, 24.4, 24.3, 21.6, 11.1; IR (thin film): 2929, 2850, 1658, 1591, 1570, 1526, 1474, 1443, 1377, 1260, 1208, 1181, 1024 cm^−1^; HRMS: Calcd. for C_24_H_32_N_4_O: 392.2576, Found: 392.2575.

*2-(N-Allyl-N-(6-methyl-2-phenylpyrimidin-4-yl)amino)-N-p-tolylmethan-acetamide *(**1e**). white solid, (498 mg, 60%), mp 124–125 °C, ^1^H-NMR: δ (ppm) 8.43–8.24 (m, 2H), 7.52–7.35 (m, 3H), 7.02 (d, 2H, *J *= 8.0 Hz), 6.94 (d, 2H, *J* = 8.0 Hz), 6.79 (br s, 1H), 6.25 (s, 1H), 5.91–5.80 (m, 1H), 5.31–5.14 (m, 2H), 4.39 (s, 2H), 4.29 (s, 2H), 4.16 (s, 2H), 2.44 (s, 3H), 2.25 (s, 3H); ^13^C-NMR: δ (ppm) 169.5, 166.4, 163.3, 162.1, 137.9, 136.9, 134.7, 131.3, 130.2, 129.2, 128.2, 128.0, 127.3, 117.8, 99.9, 52.2, 51.4, 42.9, 24.5, 21.0; IR (thin film): 2920, 2356, 1653, 1591, 1570, 1531, 1496, 1439, 1408, 1374, 1260, 1230, 1194, 1068, 1024 cm^−1^; HRMS: Calcd. for C_24_H_26_N_4_O: 386.2107, Found: 386.2104.

*2-(N-Allyl-N-(5-nitropyridin-2-yl)amino)-N-cyclohexylbutanamide* (**1f**). Yellow solid, (508 mg, 41%), mp 111–112 °C, ^1^H-NMR: δ (ppm) 9.04 (d, 1H, *J* = 2.2 Hz), 8.21 (dd, 1H, *J* = 2.2, 9.4 Hz), 6.52 (d, 1H, *J* = 9.4 Hz), 6.18 (br s, 1H), 5.87–5.72 (m, 1H), 5.38-5.11 (m, 3H), 4.23 (dd, 1H, *J* = 4.8, 17.0 Hz), 4.03 (d, 1H, *J* = 17.0 Hz), 3.78–3.66 (m, 1H), 2.19–2.03 (m, 1H), 1.96–1.83 (m, 1H), 1.80–1.49 (m, 5H), 1.43–1.22 (m, 2H), 1.21–1.06 (m, 2H), 1.05–0.88 (m, 4H); ^13^C-NMR: δ (ppm) 169.2, 160.7, 145.6, 132.9, 132.7, 126.7, 117.6, 106.7, 60.0, 48.0, 47.9, 32.9, 32.8, 25.4, 24.5, 21.9, 10.8; IR (thin film): 2924, 2850, 1657, 1588, 1570, 1496, 1413, 1325, 1286, 1251, 1111 cm^−1^; HRMS: Calcd. for C_18_H_26_N_4_O_3_: 346.2005, Found: 346.2016.

*N-(4-Chlorobenzyl)-2-(N-allyl-N-(2,6-dimethylpyrimidin-4-yl)amino)-3-methylbutanamide* (**1g**). White solid, (2.1 gm, 68%), mp 97–98 °C, ^1^H-NMR: δ (ppm) 7.23 (d, 2H, *J* = 8.2 Hz), 7.07 (d, 2H, *J* = 8.2 Hz), 6.13 (s, 1H), 5.70–5.55 (m, 1H), 5.13 (d, 2H, *J* = 13.3 Hz), 4.91 (br s, 1H), 4.40–4.25 (m, 2H), 4.10–3.90 (m, 2H), 2.56–2.42 (m, 1H), 2.38 (s, 3H), 2.33 (s, 3H), 1.02 (d, 3H, *J* = 6.4 Hz), 0.81 (d, 3H, *J* = 6.4 Hz); ^13^C-NMR: δ (ppm) 170.9, 166.1, 165.4, 162.6, 136.7, 133.1, 133.0, 128.9, 128.7, 117.4, 100.6, 42.6, 26.5, 26.1, 24.1, 19.9, 19.1; IR (thin film): 2968, 1671, 1583, 1535, 1474, 1403, 1339, 1268, 1203, 1085 cm^−1^; HRMS: Calcd. for C_21_H_27_ClN_4_O: 386.1873, Found: 386.1868.

*Ethyl 3-(7-(1-(cyclohexylcarbamoyl)propyl)-6,7-dihydro-2,4-dimethyl-5H-pyrrolo[2,3-d]pyrimidin-5-yl)propanoate *(**2a**). Yellow liquid, (200 mg, 48%), ^1^H-NMR: δ (ppm) 6.43 (d, 1H, *J* = 7.5 Hz), 4.40 (t, 1H, *J* = 7.7 Hz), 4.06 (q, 2H, *J* = 6.8 Hz), 3.80–3.63 (m, 2H), 3.40–3.25 (m, 2H), 2.45 (s, 3H), 2.34–2.20 (m, 5H), 2.10–1.95 (m, 2H), 1.90–1.70 (m, 4H), 1.69–1.46 (m, 3H), 1.40–1.05 (m, 8H), 0.88 (t, 3H, *J* = 7.3 Hz); ^13^C-NMR: δ (ppm) 172.8), 169.1, 166.3, 166.2, 157.2, 116.4, 60.6, 57.9, 50.5, 47.8, 35.0, 32.9, 32.8, 30.4, 28.9, 25.8, 25.4, 24.5, 24.4, 20.8, 20.5, 14.2, 10.7; IR (thin film): 2929, 2855, 2356, 1731, 1657, 1609, 1570, 1517, 1448, 1400, 1312, 1277, 1260, 1168, 1168, 1089, 1028 cm^−1^; HRMS: Calcd. for C_23_H_36_N_4_O_3_: 416.2787, Found: 416.2767.

*Ethyl 3-(1-(1-(cyclohexylcarbamoyl)-3-methylbutyl)-2,3-dihydro-1H-pyrrolo[3,2-c]pyridin-3-yl)-propanoate* (**2b**). Pale yellow liquid, ( 100 mg, 24%), ^1^H-NMR: δ (ppm) 8.21 (s, 1H), 8.12 (d, 1H, *J* = 5.8 Hz), 6.55 (d, 1H, *J* = 5.8 Hz), 6.04 (br s, 1H), 4.10 (q, 2H, *J* = 7.1 Hz), 3.93 (t, 1H, *J *= 6.5 Hz), 3.80–3.60 (m, 3H), 3.00–2.88 (m, 1H), 2.29 (t, 2H, *J* = 7.0 Hz), 2.02 (dd, 1H, *J* = 7.0, 13.4 Hz), 1.95–1.78 (m, 4H), 1.74–1.50 (m, 6H), 1.35–1.15 (m, 11H), 0.95–0.82 (m, 2H); ^13^C-NMR: δ (ppm) 173.0, 171.1, 148.1, 147.6, 146.1, 127.0, 107.0, 60.4, 60.2, 49.3, 48.2, 39.6, 33.7, 33.0, 32.8, 30.3, 30.0, 29.0, 25.3, 24.8, 24.7, 24.5, 22.2, 14.1; IR (thin film): 2924, 2840, 2361, 1727, 1644, 1660, 1517, 1448, 1374, 1277, 1260, 1163, 1098, 1064, 1028 cm^−1^; HRMS: Calcd. for C_24_H_37_N_3_O_3_: 415.2835, Found: 415.2837.

*Ethyl 3-(1-(1-(tert-butylcarbamoyl)-3-methylbutyl)-2,3-dihydro-1H-pyrrolo[3,2-c]pyridin-3-yl)-propanoate *(**2c**). Pale brown liquid, (85 mg, 27%), ^1^H-NMR: δ (ppm) 8.23 (s, 1H), 8.14 (d, 1H, *J* = 5.8 Hz), 6.55 (d, 1H, *J* = 5.8 Hz), 5.85 (br s, 1H), 4.10 (q, 2H, *J *= 7.1 Hz), 3.82 (t, 1H, *J *= 6.5 Hz), 3.74–3.62 (m, 2H), 3.00–2.87 (m, 1H), 2.30 (t, 2H, *J* = 7.0 Hz), 2.26–2.17 (m, 1H), 2.06–196 (m, 1H), 1.94–1.83 (m, 1H), 1.65–1.55 (m, 2H), 1.32 (s, 9H), 1.31–1.18 (m, 9H); ^13^C-NMR: δ (ppm) 173.0, 171.1, 156.2, 148.0, 146.7, 127.4, 107.0, 60.9, 60.4, 51.2, 49.4, 39.6, 33.8, 33.3, 30.3, 28.5, 24.6, 22.2, 14.2;. IR (thin film): 2960, 2933, 2361, 1727, 1666, 1596, 1543, 1505, 1448, 1365, 1277, 1255, 1220, 1163 cm^−1^; HRMS: Calcd. for C_22_H_35_N_3_O_3_: 389.2678, Found: 389.2679.

*Ethyl 3-(7-(1-(cyclohexylcarbamoyl)propyl)-6,7-dihydro-4-methyl-2-phenyl-5H-pyrrolo[2,3-d]-pyrimidin-5-yl)propanoate *(**2d**). Pale yellow liquid, (265 mg, 55%), ^1^H-NMR: δ (ppm) 8.40–8.30 (m, 2H), 7.54–7.38 (m, 3H), 6.52 (br s, 1H), 4.63–4.45 (m, 1H), 4.09 (q, 2H, *J* = 7.0 Hz), 3.80–3.60 (m, 2H), 3.50–3.25 (m, 2H), 2.40 (s, 3H), 2.37–2.24 (m, 2H), 2.18–1.96 (m, 2H), 1.94–1.70 (m, 4H), 1.63–1.38 (m, 3H), 1.34–1.14 (m, 6H), 1.05–0.80 (m, 5H); ^13^C-NMR: δ (ppm) 172.8, 169.3, 166.6, 163.0, 157.9, 138.2, 130.0, 128.2, 127.7, 117.5, 60.6, 58.2, 50.7, 47.9, 35.2, 32.8, 32.7, 30.4, 28.8, 25.3, 24.4, 24.2, 21.0, 20.9, 14.2, 10.8; IR (thin film): 2972, 2929, 2850, 1731, 1662, 1605, 1562, 1531, 1452, 1377, 1312, 1246, 1163 cm^−1^, HRMS: Calcd. for C_28_H_38_N_4_O_3_: 478.2944, Found: 478.2950.

*N-Cyclohexyl-2-(5,6-dihydro-4-methyl-5-(3-oxobutyl)-2-phenylpyrrolo[2,3-d]pyrimidin-7-yl)butan-amide *(**2e**). Yellow liquid, (75 mg, 44%), ^1^H-NMR: δ (ppm) 8.40–8.25 (m, 2H), 7.55–7.35 (m, 3H), 6.50 (d, 1H, *J* = 8.0Hz), 4.50 (t, 1H, *J* = 6.6 Hz), 3.78–3.58 (m, 2H), 3.45–3.25 (m, 2H), 2.47 (t, 2H, *J* = 7.5 Hz), 2.40 (s, 3H), 2.16–2.00 (m, 5H), 1.90–1.66 (m, 4H), 1.60–1.34 (m, 4H), 1.30–1.14 (m, 2H), 1.10–0.85 (m, 5H); ^13^C-NMR: δ (ppm) 207.9, 169.3, 166.5, 162.9, 157.8, 138.1, 130.0, 128.2, 127.7, 117.7, 58.2, 51.2, 47.8, 39.4, 35.1, 32.7, 30.0, 27.4, 25.3, 24.4, 24.3, 21.1, 21.0, 10.8; IR (thin film): 2929, 2853, 1710, 1656, 1603, 1564, 1508, 1454, 1377, 1317, 1247, 1159, 1064 cm^−1^, HRMS: Calcd. for C_27_H_36_N_4_O_2_: 448.2838, Found: 448.2836.

*Ethyl 3-(7-(N-p-tolylmethan-carbamoylmethyl)-6,7-dihydro-4-methyl-2-phenyl-5H-pyrrolo[2,3-d]-pyrimidin-5-yl)propanoate *(**2f**). Pale yellow liquid, (125 mg, 27%), ^1^H-NMR: δ (ppm) 8.28 (d, 2H, *J* = 7.6 Hz), 7.45–7.34 (m, 3H), 7.10 (d, 2H, *J* = 7.6 Hz), 7.00 (d, 2H, *J* = 7.6 Hz), 6.95 (br s, 1H), 4.50–4.25 (m, 3H), 4.08–3.95 (m, 3H), 3.75–3.65 (m, 1H), 3.45–3.35 (m, 2H), 2.40 (s, 3H), 2.33–2.20 (m, 5H), 2.10–2.00 (m, 1H), 1.96–1.85 (m, 1H), 1.20 (t, 3H, *J* = 7.0 Hz); ^13^C-NMR: δ (ppm) 173.0, 169.0, 167.0, 163.6, 158.5, 138.0, 137.1, 135.0, 130.1, 129.4, 128.3, 128.0, 127.6, 117.4, 60.8, 55.8, 49.5, 43.2, 35.8, 30.8, 28.7, 21.1, 21.0, 14.2; IR (thin film): 2924, 2356, 1731, 1662, 1605, 1583, 1570, 1518, 1448, 1377, 1325, 1260, 1172, 1024 cm^−1^, HRMS: Calcd. for C_28_H_32_N_4_O_3_: 472.2474, Found : 472.2473.

*Ethyl 3-(1-(1-(cyclohexylcarbamoyl)propyl)-2,3-dihydro-5-nitro-1H-pyrrolo[2,3-b]pyridin-3-yl)-propanoate *(**2g**). Pale brown liquid, (297 mg, 48%), ^1^H-NMR: δ (ppm) 8.86 (s, 1H), 7.92 (s, 1H), 6.04 (br s, 1H), 4.05 (t, 1H, *J* = 7.5 Hz), 4.15 (q, 2H, *J* = 7.2 Hz), 4.00–3.83 (m, 1H), 3.79–3.62 (m, 1H), 3.55–3.30 (m, 2H), 2.44–2.30 (m, 2H), 2.20–1.98 (m, 2H), 1.94–1.76 (m, 3H), 1.74–1.64 (m, 3H), 1.62–1.50 (m, 2H), 1.34–1.25 (m, 6H), 1.20–1.10 (m, 1H), 0.95 (t, 3H, *J* = 7.2 Hz); ^13^C-NMR: δ (ppm) 172.5, 168.1, 164.6, 146.7, 136.6, 126.7, 125.7, 60.8, 58.5, 51.9, 48.2, 36.2, 33.0, 31.0, 30.9, 29.5, 25.4, 24.6, 24.5, 21.4, 14.3, 10.7; I.R. (thin film): 2933, 2850, 2356, 1731, 1657, 1609, 1574, 1517, 1496, 1443, 1377, 1291, 1186, 1090, 1024 cm^−1^, HRMS: Calcd. for C_22_H_32_N_4_O_5_: 432.2373, Found: 432.2386.

*N-Cyclohexyl-2-(5,6-dihydro-2,4-dimethyl-5-(3-oxo-3-phenylpropyl)pyrrolo[2,3-d]pyrimidin-7-yl)-butanamide *(**2h**). Pale brown liquid, (150 mg, 34%), ^1^H-NMR: δ (ppm) 7.90 (d, 2H, *J* = 7.8 Hz), 7.58–7.54 (m, 1H), 7.48–7.41 (m, 2H), 6.48 (d, 1H, *J* = 7.8 Hz), 4.41 (t, 1H, *J* = 7.7 Hz), 3.80–3.65 (m, 2H), 3.46–3.30 (m, 2H), 3.08–2.83 (m, 2H), 2.45 (s, 3H), 2.30 (s, 3H), 2.26–2.14 (m, 1H), 2.08–1.96 (m, 1H), 1.94–1.40 (m, 7H), 1.30–0.98 (m, 5H), 0.90 (t, 3H, *J* = 7.3 Hz); ^13^C-NMR: δ (ppm) 199.3, 169.2, 166.3, 166.1, 157.2, 136.6, 133.2, 128.6, 127.9, 116.8, 58.0, 50.7, 47.9, 35.2, 34.1, 33.0, 32.8, 28.2, 25.8, 25.4, 24.4, 21.1, 20.6, 10.8; IR (thin film): 2929, 2853, 1671, 1611, 1568, 1515, 1446, 1404, 1358, 1317, 1270, 1259, 1202, 1083 cm^−1^, HRMS: Calcd. for C_27_H_36_N_4_O_2_: 448.2838, Found: 448.2843.

*Ethyl 3-(7-(1-(4-chlorobenzylcarbamoyl)-2-methylpropyl)-6,7-dihydro-2,4-dimethyl-5H-pyrrolo[2,3-d]pyrimidin-5-yl)propanoate* (**2i**). Pale yellow liquid, (203 mg, 43%), ^1^H-NMR: δ (ppm) 7.22 (d, 2H, *J* = 8.0 Hz), 7.07 (d, 2H, *J *= 8.0 Hz), 6.97 (d, 1H, *J* = 6.0 Hz), 4.39 (dd, 2H, *J* = 6.0, 14.7 Hz), 4.12 (q, 2H, *J* = 7.0 Hz), 4.04–3.97 (m, 1H), 3.73 (t, 1H, *J* = 7.0 Hz), 3.40–3.20 (m, 2H), 2.51–2.38 (m, 1H), 2.35 (s, 3H), 2.28 (s, 3H), 2.23 (t, 2H, *J* = 7.5 Hz), 2.10–1.90 (m, 1H), 1.80–1.65 (m, 1H), 1.25 (t, 3H, *J* = 7.0 Hz), 1.00 (d, 3H, *J* = 7.0 Hz), 0.87 (d, 3H, *J* = 7.0 Hz); ^13^C-NMR: δ (ppm) 172.8, 169.6, 166.1, 165.9, 157.3, 136.7, 133.2, 128.9, 128.7, 116.6, 64.0, 60.6, 51.5, 42.6, 35.2, 30.6, 28.9, 26.4, 25.7, 20.6, 19.5, 19.1, 14.2; IR (thin film): 2964, 2924, 1731, 1671, 1609, 1570, 1531, 1469, 1403, 1273, 1177, 1094, 1011 cm^−1^, HRMS: Calcd. for C_25_H_33_ClN_4_O_3_: 472.2241, Found : 472.2244.

## 4. Conclusions

The reported two-step cascade constitutes a concise entry into pyrrolidino- pyridines and pyrimidines. Compared with previous results obtained on xanthate cascades performed on *N*-allylamino- pyridine and pyrimidines, these new couplings significantly widen the scope of these reactions. The modest efficiency of the whole process is counter-balanced by the simple experimental procedure and the straightforward access to important biologically relevant scaffolds [40,41,42,43]. This work is a new example of the potential of radical chemistry in Ugi post-condensations.
